# A New Scoring System for Predicting In-hospital Death in Patients Having Liver Cirrhosis With Esophageal Varices

**DOI:** 10.3389/fmed.2021.678646

**Published:** 2021-10-11

**Authors:** Fengshuo Xu, Luming Zhang, Zichen Wang, Didi Han, Chengzhuo Li, Shuai Zheng, Haiyan Yin, Jun Lyu

**Affiliations:** ^1^Department of Clinical Research, The First Affiliated Hospital of Jinan University, Guangzhou, China; ^2^School of Public Health, Xi'an Jiaotong University Health Science Center, Xi'an, China; ^3^Intensive Care Unit, The First Affiliated Hospital of Jinan University, Guangzhou, China; ^4^Department of Public Health, University of California, Irvine, Irvine, CA, United States; ^5^School of Public Health, Shaanxi University of Chinese Medicine, Xianyang, China

**Keywords:** liver cirrhotic with esophageal varices, MIMIC, nomogram, prognosis, in-hospital death

## Abstract

**Introduction:** Liver cirrhosis is caused by the development of various acute and chronic liver diseases. Esophageal varices is a common and serious complication of liver cirrhosis during decompensation. Despite the development of various treatments, the prognosis for liver cirrhosis with esophageal varices (LCEV) remains poor. We aimed to establish and validate a nomogram for predicting in-hospital death in LCEV patients.

**Methods:** Data on LCEV patients were extracted from the Medical Information Mart for Intensive Care III and IV (MIMIC-III and MIMIC-IV) database. The patients from MIMIC-III were randomly divided into training and validation cohorts. Training cohort was used for establishing the model, validation and MIMIC-IV cohorts were used for validation. The independent prognostic factors for LCEV patients were determined using the least absolute shrinkage and selection operator (LASSO) method and forward stepwise logistic regression. We then constructed a nomogram to predict the in-hospital death of LCEV patients. Multiple indicators were used to validate the nomogram, including the area under the receiver operating characteristic curve (AUC), calibration curve, Hosmer-Lemeshow test, integrated discrimination improvement (IDI), net reclassification index (NRI), and decision curve analysis (DCA).

**Results:** Nine independent prognostic factors were identified by using LASSO and stepwise regressions: age, Elixhauser score, anion gap, sodium, albumin, bilirubin, international normalized ratio, vasopressor use, and bleeding. The nomogram was then constructed and validated. The AUC value of the nomogram was 0.867 (95% CI = 0.832–0.904) in the training cohort, 0.846 (95% CI = 0.790–0.896) in the validation cohort and 0.840 (95% CI = 0.807–0.872) in the MIMIC-IV cohort. High AUC values indicated the good discriminative ability of the nomogram, while the calibration curves and the Hosmer-Lemeshow test results demonstrated that the nomogram was well-calibrated. Improvements in NRI and IDI values suggested that our nomogram was superior to MELD-Na, CAGIB, and OASIS scoring system. DCA curves indicated that the nomogram had good value in clinical applications.

**Conclusion:** We have established the first prognostic nomogram for predicting the in-hospital death of LCEV patients. The nomogram is easy to use, performs well, and can be used to guide clinical practice, but further external prospective validation is still required.

## Introduction

Liver cirrhosis is a chronic liver disease characterized by pseudolobule formation, hepatocyte necrosis, regenerated nodules, and diffused fibrosis. It is caused by advanced liver disease with a complex clinical pathogenesis. Most scholars believe that it is related to liver damage caused by bile acid deposition, immune factors, alcohol, viruses, and other long-term ongoing effects (1). Portal hypertension and liver function injury are the main manifestations of advanced liver cirrhosis, while esophageal varices is one of the most serious complications of portal hypertension in liver cirrhosis. Reportedly 30–70% of liver cirrhosis patients develop esophageal varices, and 5–15% will experience rupture bleeding, with mortality occurring in up to 30% of cases of the first hemorrhage (2, 3). Patients having liver cirrhosis with esophageal varices (LCEV) are also prone to acute chronic liver failure, hepatorenal syndrome, ascites (AC), hepatic encephalopathy (HE), and other complications (4, 5).

The improvements in quality of life and an increasingly aging society are increasing the incidence of LCEV, and it is therefore urgent for effective clinical treatments to be identified. Current first-line treatments include vasoactive drugs, prophylactic antibiotics, and endoscopic vein ligation. Despite improvements in diagnosis and treatment, mortality rates in LCEV patients remain high, with rates of 13.4–22.7% at 6 weeks (6–9). It is therefore critical to develop a severity scoring system stratified by mortality risk to accurately and rapidly assess the prognosis and guide treatments in individual LCEV patients

Many existing scoring systems have been used to evaluate the prognosis of LCEV patients, but none of them are targeted. These scoring systems can be divided into two types. One type focuses on assessing the prognosis of patients with liver cirrhosis, including the Model for End-Stage Liver Disease (MELD), Child-Pugh score, and MELD-Na, which add serum sodium to the MELD system (10, 11). The other type evaluates acute upper gastrointestinal bleeding, including the Glasgow Blatchford, Rockall, and AIMS65 (12). Bai et al. recently proposed the cirrhosis acute gastrointestinal bleeding (CAGIB) system, which includes diabetes (DB), hepatocellular carcinoma (HCC), bilirubin, albumin, alanine aminotransferase (ALT), and creatinine (13). However, the prognostic value for LCEV patients of these scoring systems is very limited (2).

The main objective of this study was therefore to identify the significant prognostic factors for LCEV patients from a large database, and to establish and validate an easy-to-use prognostic nomogram that predicts their in-hospital death. The nomogram will help clinicians to stratify the risk of LCEV patients and develop treatment strategies, and also help the families of patients to understand their condition.

## Methods

### Data Source

LCEV patient data were obtained from Medical Information Mart for Intensive Care III and IV (MIMIC-III v1.4 and MIMIC-IV v1.0) database. MIMIC is a large, single-center, open-access database. MIMIC-III includes data on more than 58,000 admissions to Beth Israel Deaconess Medical Center in Boston from 2001 to 2012, comprising 38,645 adults and 7,875 newborns (14–16)And MIMIC-IV covers 524,740 admissions for 382,278 patients to this center from 2008 to 2019 (17, 18). The relevant records include demographic data, hourly vital signs, laboratory test results, microbial culture results, imaging data, treatment procedures, medication records, and survival information.

The use of the MIMIC-III and MIMIC-IV databases was approved by the Institutional Review Board of the Beth Israel Deaconess Medical Center and Massachusetts Institute of Technology, and all patient information in the database is anonymous, so informed consent was not required (19, 20).

We completed the online course and examination to gain access to the database (Record ID: 38455175).

### Patients and Variables

We used SQL (Structured Query Language) programming in Navicat Premium (version 11.2.7.0) to extract data. ICD-9 (ninth edition of the International Classification of Diseases) codes were used to identify LCEV patients: codes 5712, 5715, and 5716 for liver cirrhosis; and codes 4560, 4561, 45620, and 45621 for esophageal varices. The exclusion criteria were aged <18 or >89 years, or dying within 24 h of admission to an intensive care unit (ICU). Patient data for the first admission only were used for those who had been admitted multiple times to the ICU.

After identifying eligible subjects, we used their hadm_id and icustay_id parameters to extract information from the corresponding tables, including age, gender, marital status, ethnicity, insurance, comorbidities, 24-h urine output, vital signs, laboratory parameters, renal replacement treatment (RRT)use, mechanical ventilation (Mechvent) use, vasopressor use, severity scoring system, and survival information. Comorbidities included HE, AC, HCC, DB, and the Elixhauser score. The vital signs used were the mean values during the first 24 h of the ICU stay, including heart rate, mean blood pressure (MBP), respiratory rate, temperature, and percutaneous oxygen saturation (SpO_2_). The laboratory parameters analyzed were those that were first obtained after the ICU admission. The study indexes were ALT, aspartate aminotransferase (AST), albumin, bilirubin, alkaline phosphtase (AP), anion gap (AG), bicarbonate, phosphate, chloride, calcium, magnesium, potassium, sodium, glucose, lactate dehydrogenase (LD), creatinine, blood urea nitrogen (BUN), hematocrit, hemoglobin, mean corpuscular hemoglobin (MCH), mean corpuscular volume (MCV), red blood cell distribution width (RDW), red blood cells (RBC), white blood cells (WBC), platelet, international normalized ratio (INR), prothrombin time (PT), and partial prothrombin time (PTT). Severity scoring systems included the Glasgow Coma Scale (GCS) and Oxford Acute Severity of Illness Score (OASIS).

Marital status was classified into married, unmarried, and other (divorced, separated, or widowed). Race categories were white, black, and other. We also classified liver cirrhosis into two categories of etiology (cholestasis or alcoholic, and other) and classified esophageal varices into bleeding and not-bleeding categories.

The MELD-Na and CAGIB scores were calculated using relevant data in the following formula: MELD-Na = 3.8 × log_e_(bilirubin [mg/dl]) + 11.2 × log_e_(INR) + 9.6 × log_e_(creatinine [mg/dl]) + 6.4 × (etiology: 0 for cholestasis or alcohol, otherwise 1) + 1.59 × [135 – sodium (mmol/L)], and CAGIB score = DB(1 for yes, 0 for no) × 1.040 + HCC(1 for yes, 0 for no) × 0.974 + bilirubin (μmoI/L) × 0.005 – albumin (g/L) × 0.091 + ALT (U/L) × 0.001 + creatinine (μmoI/L) × 0.012 – 3.964 (21).

The endpoint for our study was in-hospital death. Patients who were still alive at discharge were designated as alive.

### Statistical Analysis

Missing data are common in the MIMIC database, and this study used multiple imputation to account for missing data. And in order to avoid excessive bias, the missing proportion of variables studied in this research was <20%. Multiple imputation technique involves creating multiple copies of the data and replacing the missing values by selecting a suitable random sample from the predicted distribution (14). We used the mice package of R software to obtain 10 estimated data sets. Predictive mean matching and logistic regression methods were used for continuous and categorical variables, respectively. The specific missing proportion of variables before imputation is shown in Supplementary Figure 1.

We randomly assigned 70% of patients in MIMIC-III database to the training cohort and 30 % to the validation cohort. The training cohort was used to establish the nomogram, while the validation cohort and MIMIC-IV cohort were used to perform validation. Frequency and percentage was used to describe the categorical variables, and the chi-square test or Fisher's exact test was used to identify differences between groups. The Shapiro-Wilk test was applied to continuous variables to confirm that they conformed to a normal distribution. Those that did were described using mean and standard-deviation values, and a Student's *t*-test was used to identify differences between groups. The other continuous variables were described using median and interquartile-range (IQR), and the Mann-Whitney *U*-test was used to identify differences between groups.

Logistic regression was used to identify risk factors that were independently associated with the in-hospital death of LCEV patients (OASIS, MELD-Na, and CAGIB systems were not included in the analysis). Because of the large number of variables in our study, we used two steps to screen for independent prognostic factors. We first used the least absolute shrinkage and selection operator (LASSO) method for conducting preliminary screening to solve the collinearity effect. The LASSO method reduces the coefficient of irrelevant variables to zero, while retaining important variables (22). The largest value of lambda was chosen when the cross-validation error was within one standard error of the minimum. The variables selected by LASSO were then further screened using the forward LN stepwise regression method. The probability threshold was 0.05 for entry and 0.10 for removal. All identified independent prognostic factors were used to establish a logistic regression model and the results were presented as odds ratios (ORs) and 95% confidence intervals (CIs). Collinearity between continuous variables was tested by the variance inflation factor (VIF), and an arithmetic square root of VIF ≤ 2 was considered as non-collinearity (23). Finally, we established a nomogram that included all independent prognostic factors that predict in-hospital death in LCEV patients. We also constructed a dynamic nomogram using the DynNom package of R software to facilitate the application of the new model.

The nomograms were validated using multiple indicators. The area under the receiver operating characteristic curve (AUC) assessed the discriminative ability of the nomogram, which was compared with the AUC values of the OASIS, MELD-Na, and CAGIB systems. The receiver operating characteristic curve was used to determine the optimal cutoff value and its corresponding sensitivity and specificity according to Youden's index. The integrated discrimination improvement (IDI) and the net reclassification index (NRI) were also used to calculate how the performance of the nomogram improves on the other scoring systems. We further plotted calibration curves and performed Hosmer-Lemeshow test to evaluate the calibration of the nomogram. Decision curve analysis (DCA) was used to evaluate the net benefits of medical interventions under the guidance of the nomogram and the OASIS, MELD-Na, and CAGIB systems. We also performed a subgroup analysis to evaluate the application of the nomogram in the bleeding and non-bleeding cohorts via AUC, *P* < 0.05 were considered statistically significant. R software (version 4.0.3) and SPSS software (version 24.0) were used for all analyses. The R packages used included glmnet, lattice, MASS, nnet, mice, rms, foreign, regplot, pROC, nricens, PredictABEL, DynNom, survival, and reconnect.

## Results

### Baseline Characteristics

After applying the inclusion and exclusion criteria, 813 LCEV patients were identified from MIMIC-III database (569 and 244 in the training and validation cohorts, respectively) and 930 LCEV patients were identified from MIMIC-IV database. Among the causes of liver cirrhosis, the rates of alcohol or cholestasis were 54.7 and 54.9%, respectively, in the training and validation cohorts. Bleeding from esophageal varices (41.7 and 44.3% in the training and validation cohorts) was slightly less common than not bleeding. There were fewer patients with HE (22.1 and 23.0% in the training and validation cohorts, respectively), AC (29.9 and 32.4%), HCC (12.0 and 14.3%), and DB (28.6 and 31.1%). The median ages of patients in the training and validation cohorts were 54 years (IQR 48–62 years) and 56.5 years (IQR 51–64 years), respectively. Most patients were male (68.7 and 70.1% in the training and validation cohorts, respectively), married (42.5 and 42.6%), and white (75.9 and 74.2%). The remaining baseline characteristics of the patients are listed in Table 1. None of the continuous variables in this study were normally distributed. All characteristics except for AP were evenly distributed across the training and validation cohorts. The characteristics of the patients from the MIMIC-IV database are shown in Supplementary Table 1. The in-hospital death rates in the MIMIC-III and MIMIC-IV cohorts were 18.7 and 16.7%, respectively.

**Table 1:** Baseline demographic and laboratory characteristics of LCEV patients in MIMIC-III database.

**Variables**	**Total cohort**	**Training cohort**	**Validation cohort**	***P*** **-value**
*N*	813	569	244	
Cause, *n* (%)				0.945
Cholestasis or alcoholic	445 (54.7)	311 (54.7)	134 (54.9)	
Other	368 (45.3)	258 (45.3)	110 (45.1)	
Bleeding, *n* (%)				0.490
No	468 (57.6)	332 (58.3)	136 (55.7)	
Yes	345 (42.4)	237 (41.7)	108 (44.3)	
HE, *n* (%)				0.800
No	631 (77.6)	443 (77.9)	188 (77.0)	
Yes	182 (22.4)	126 (22.1)	56 (23.0)	
AC, *n* (%)				0.478
No	564 (69.4)	399 (70.1)	165 (67.6)	
Yes	249 (30.6)	170 (29.9)	79 (32.4)	
HCC, *n* (%)				0.347
No	710 (87.3)	501 (88.0)	209 (85.7)	
Yes	103 (12.7)	68 (12.0)	35 (14.3)	
DB, *n* (%)				0.473
No	574 (70.6)	406 (71.4)	168 (68.9)	
Yes	239 (29.4)	163 (28.6)	76 (31.1)	
Age (Year) (median [IQR])	55.0 [49.0,63.0]	54.0 [48.0,62.0]	56.5 [51.0,64.0]	0.007
Gender, *n* (%)				0.699
Male	562 (69.1)	391 (68.7)	171 (70.1)	
Female	251 (30.9)	178 (31.3)	73 (29.9)	
Marrital Status, *n* (%)				0.988
Married	346 (42.6)	242 (42.5)	104 (42.6)	
Unmarried	306 (37.6)	215 (37.8)	91 (37.3)	
Other	161 (19.8)	112 (19.7)	49 (20.1)	
Ethnicity, *n* (%)				0.595
White	613 (75.4)	432 (75.9)	181 (74.2)	
Black	77 (9.5)	50 (8.8)	27 (11.1)	
Other	123 (15.1)	87 (15.3)	36 (14.8)	
Insurance, *n* (%)				0.868
Government	34 (4.2)	25 (4.4)	9 (3.7)	
Medicaid	179 (22.0)	126 (22.1)	53 (21.7)	
Medicare	277 (34.1)	195 (34.3)	82 (33.6)	
Private	312 (38.4)	214 (37.6)	98 (40.2)	
Self-pay	11 (1.4)	9 (1.6)	2 (0.8)	
Heart rate (min^−1^) (median [IQR])	83.5 [71.1,95.6]	83.1 [70.8,95.4]	84.7 [71.3,95.6]	0.402
MBP (mmHg) (median [IQR])	75.2 [68.6,83.9]	75.0 [68.8,84.0]	75.4 [68.3,83.6]	0.954
Respiratory rate (min^−1^) (median [IQR])	16.9 [14.8,19.4]	17.0 [14.8,19.5]	16.8 [14.8,19.0]	0.635
Temperature (°C) (median [IQR])	36.7 [36.3,37.0]	36.7 [36.3,37.0]	36.6 [36.3,37.0]	0.986
SpO_2_ (%) (median [IQR])	97.7 [96.3,98.9]	97.6 [96.2,98.8]	97.8 [96.6,98.9]	0.222
24-h urine output (mL) (median [IQR])	1258.0 [725.0,2010.0]	1266.0 [725.0,1995.0]	1240.0 [707.0,2028.5]	0.669
ALT (IU/L) (median [IQR])	32.0 [21.0,67.0]	33.0 [22.0,67.0]	32.0 [21.0,65.5]	0.840
AST (IU/L) (median [IQR])	67.0 [41.0,131.0]	67.0 [41.0,131.0]	64.0 [41.0,132.2]	0.683
Albumin (g/dL) (median [IQR])	2.8 [2.5,3.2]	2.8 [2.5,3.2]	2.8 [2.5,3.3]	0.830
Bilirubin (mg/dL) (median [IQR])	3.1 [1.6,6.0]	3.1 [1.6,6.0]	3.0 [1.6,6.0]	0.916
AP (IU/L) (median [IQR])	92.0 [67.0,133.0]	90.0 [66.0,129.0]	101.0 [69.0,152.8]	0.008
AG (mEq/L) (median [IQR])	13.0 [11.0,17.0]	13.0 [11.0,17.0]	13.0 [11.0,16.0]	0.459
Bicarbonate (mEq/L) (median [IQR])	22.0 [19.0,25.0]	22.0 [19.0,25.0]	22.0 [19.0,25.0]	0.956
Phosphate (mg/dL) (median [IQR])	3.5 [2.9,4.3]	3.6 [2.9,4.4]	3.5 [2.9,4.3]	0.491
Chloride (mEq/L) (median [IQR])	106.0 [102.0,110.0]	106.0 [102.0,110.0]	106.0 [102.0,110.0]	0.809
Calcium (mg/dL) (median [IQR])	8.0 [7.6,8.7]	8.0 [7.6,8.7]	8.0 [7.4,8.6]	0.055
Magnesium (mg/dL) (median [IQR])	1.9 [1.6,2.1]	1.9 [1.6,2.1]	1.9 [1.6,2.1]	0.763
Potassium (mEq/L) (median [IQR])	4.2 [3.8,4.7]	4.2 [3.8,4.7]	4.2 [3.8,4.7]	0.556
Sodium (mEq/L) (median [IQR])	138.0 [135.0,141.0]	138.0 [134.0,141.0]	138.0 [135.0,141.0]	0.899
Glucose (mg/dL) (median [IQR])	243.0 [190.0,322.0]	245.0 [195.0,325.0]	231.5 [186.8,314.0]	0.220
LD (IU/L) (median [IQR])	124.0 [101.0,162.0]	125.0 [101.0,166.0]	120.5 [100.0,152.0]	0.183
Creatinine (mg/dL) (median [IQR])	1.0 [0.7,1.7]	1.0 [0.7,1.7]	1.0 [0.7,1.6]	0.634
BUN (mg/dL) (median [IQR])	24.0 [16.0,44.0]	25.0 [17.0,44.0]	24.0 [16.0,43.0]	0.684
Hematocrit (%) (median [IQR])	28.3 [25.3,31.5]	28.3 [25.2,31.4]	28.4 [25.7,31.7]	0.527
Hemoglobin (g/dL) (median [IQR])	9.8 [8.6,10.8]	9.8 [8.6,10.8]	9.9 [8.6,10.8]	0.857
MCH (pg) (median [IQR])	31.7 [30.1,33.6]	31.8 [30.1,33.6]	31.5 [30.2,33.5]	0.609
MCV (fL) (median [IQR])	92.0 [88.0,99.0]	93.0 [88.0,99.0]	92.0 [88.0,98.0]	0.993
RDW (%) (median [IQR])	17.1 [15.8,18.9]	17.2 [15.8,19.1]	17.0 [15.9,18.5]	0.367
RBC (m/uL) (median [IQR])	3.1 [2.7,3.4]	3.1 [2.7,3.4]	3.1 [2.7,3.5]	0.646
WBC (k/uL) (median [IQR])	7.3 [4.6,11.2]	7.1 [4.6,11.5]	7.6 [4.8,10.5]	0.720
Platelet (k/uL) (median [IQR])	93.0 [63.0,127.0]	93.0 [62.0,126.0]	92.0 [63.0,128.5]	0.941
INR (median [IQR])	1.6 [1.4,1.9]	1.6 [1.4,2.0]	1.6 [1.4,1.9]	0.297
PT (s) (median [IQR])	17.1 [15.3,20.1]	17.2 [15.2,20.2]	16.8 [15.4,19.4]	0.333
PTT (s) (median [IQR])	36.0 [31.7,44.0]	36.1 [32.0,44.0]	35.8 [31.4,44.1]	0.794
RRT, *n* (%)				0.207
No	767 (94.3)	533 (93.7)	234 (95.9)	
Yes	46 (5.7)	36 (6.3)	10 (4.1)	
Mechvent, *n* (%)				0.654
No	496 (61.0)	350 (61.5)	146 (59.8)	
Yes	317 (39.0)	219 (38.5)	98 (40.2)	
Vasopressor, *n* (%)				0.526
No	628 (77.2)	443 (77.9)	185 (75.8)	
Yes	185 (22.8)	126 (22.1)	59 (24.2)	
Elixhauser (median [IQR])	17.0 [13.0,22.0]	16.0 [12.0,21.0]	17.0 [13.0,22.0]	0.361
GCS (median [IQR])	15.0 [14.0,15.0]	15.0 [14.0,15.0]	15.0 [14.0,15.0]	0.285
OASIS (median [IQR])	31.0 [25.0,38.0]	30.0 [24.0,37.0]	32.0 [25.0,39.0]	0.076
MELD-Na (median [IQR])	14.5 [6.4,26.5]	14.5 [5.9,27.0]	14.6 [6.9,24.8]	0.865
CAGIB (median [IQR])	−4.1 [−4.2, −3.1]	−4.1 [−4.2, −3.1]	−4.0 [−4.2, −3.1]	0.577
In-hospital death, *n* (%)				0.640
Alive	661 (81.3)	465 (81.7)	196 (80.3)	
Dead	152 (18.7)	104 (18.3)	48 (19.7)	

*LCEV, liver cirrhosis with esophageal; HE, hepatic encephalopathy; AC, ascites; HCC, hepatocellular carcinoma; DB, diabetes; IQR, interquartile-range; MBP, mean blood pressure; SpO_2_, percutaneous oxygen saturation; ALT, alanine aminotransferase; AP, alkaline phosphtaase; AG, anion gap; AST, aspartate aminotransferase; LD, lactate dehydrogenase; BUN, blood urea nitrogen; MCH, mean corpuscular hemoglobin; MCV, mean corpuscular volume; RBC, red blood cells; WBC, white blood cells; RDW, RBC distribution width; INR, international normalized ratio; PT, prothrombin time; PTT, partial prothrombin time; RRT, renal replacement treatment; Mechvent, mechanical ventilation; GCS, Glasgow Coma Scale; OASIS, Oxford Acute Severity of Illness Score; MELD-Na, Model for End-Stage Liver Disease-Na; CAGIB, cirrhosis acute gastrointestinal bleeding*.

### Nomogram Construction

**Figure 1:**
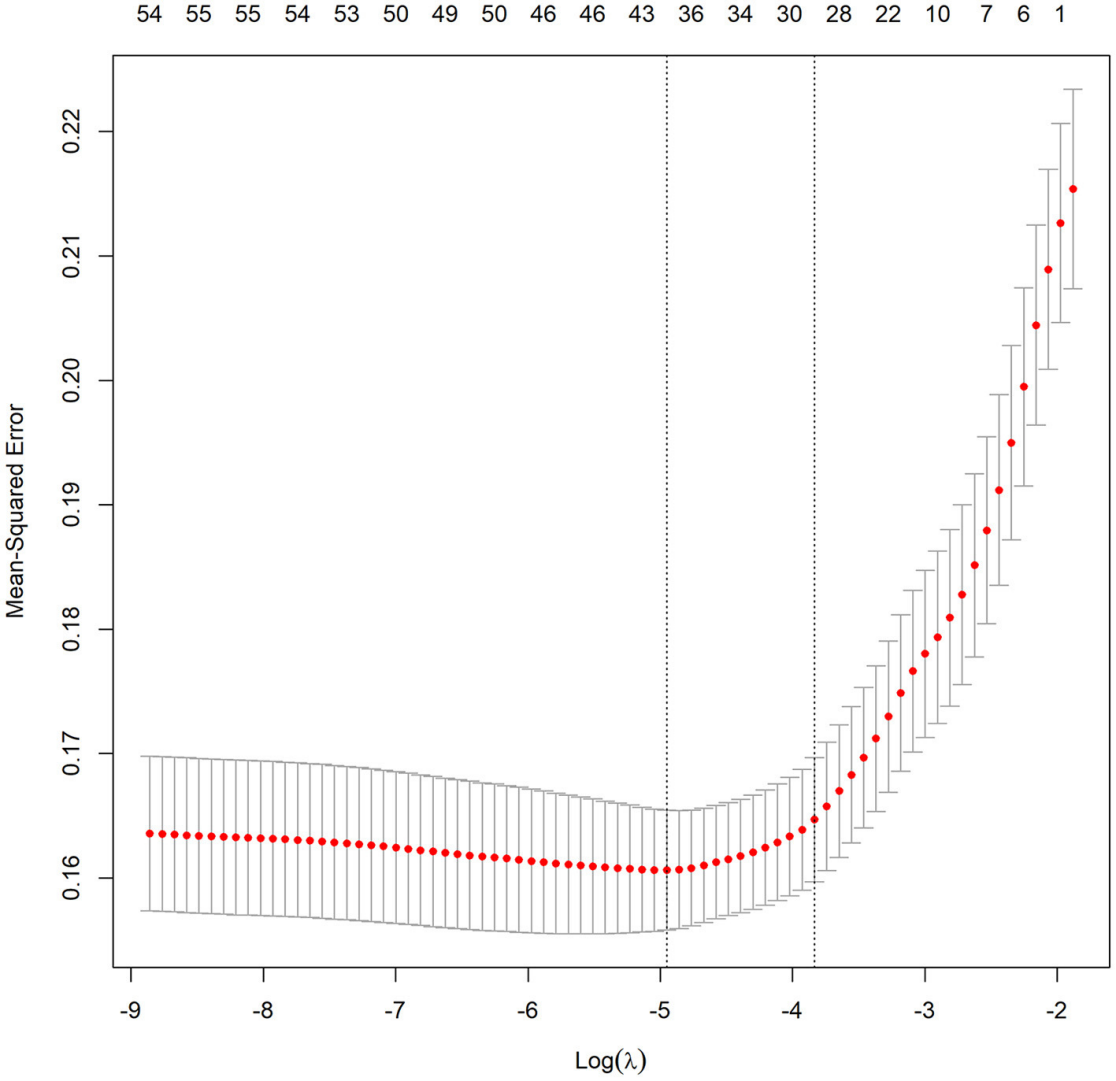
Different mean-squared error across the range of lambda. The mean-squared error was estimated with cross-validation technique and the largest lambda value was chosen when the cross-validation error was within one standard error of the minimum.

To construct the nomogram, the variables were first preliminarily screened using LASSO. Figure 1 shows the different mean-squared error within the range of log(lambda). When the cross-validation error was less than the standard error of the minimum value, the maximum lambda value was selected. The model retained 29 dummy variables: cause, bleeding, HCC, age, marital status, Insurance, heart rate, MBP, temperature, SpO_2_, urine output, albumin, bilirubin, AP, AG, bicarbonate, magnesium, potassium, sodium, LD, BUN, MCV, RDW, WBC, INR, PTT, vasopressor use, Elixhauser score, and GCS score. These variables were rescreened using forward LN stepwise regression. Independent prognostic factors were then identified, which included Age, Elixhauser score, AG, sodium, albumin, bilirubin, INR, vasopressor use, and bleeding. Their OR and 95% CI values are listed in Table 2. The VIF was calculated, and no continuous variables mentioned above had an arithmetic square root of VIF ≤ 2, indicating that collinearity was not existed in the regression model.

**Table 2:** Factors independently associated with in-hospital death in LCEV patients.

**Variables**	**OR**	**95%CI**	***P*** **-value**
Age	1.025	(1.000–1.050)	0.047
Elixhauser	1.056	(1.015–1.098)	0.007
AG	1.087	(1.026–1.152)	0.005
Sodium	0.951	(0.907–0.997)	0.036
Albumin	0.559	(0.366–0.854)	0.007
Bilirubin	1.046	(1.016–1.078)	0.002
INR	2.119	(1.444–3.109)	<0.001
Vasopressor			<0.001
No	Reference		
Yes	5.267	(2.996–9.260)	
Bleeding			0.001
No	Reference		
Yes	2.581	(1.492–4.467)	

*LCEV, liver cirrhosis with esophageal; OR, odds ratio; CI, confidence interval; AG, anion gap; INR, international normalized ratio*.

The risk of in-hospital death was 5.267-fold (OR = 5.267, 95% CI = 2.996–9.260) higher in patients who received vasopressors. The in-hospital death was 2.581 times (OR = 2.581, 95% CI = 1.492–4.467) higher in patients with esophageal varicose bleeding. The Age (OR = 1.025, 95% CI = 1.000–1.050), Elixhauser score (OR = 1.056, 95% CI = 1.015–1.098), AG (OR = 1.087, 95% CI = 1.026–1.152), bilirubin (OR = 1.046, 95% CI = 1.016–1.078), and INR (OR = 2.119, 95% CI = 1.444–3.109) were risk factors for in-hospital death, while sodium (OR = 0.951, 95% CI = 0.907–0.997) and albumin (OR = 0.559, 95% CI = 0.366–0.854) were protective factors.

**Figure 2:**
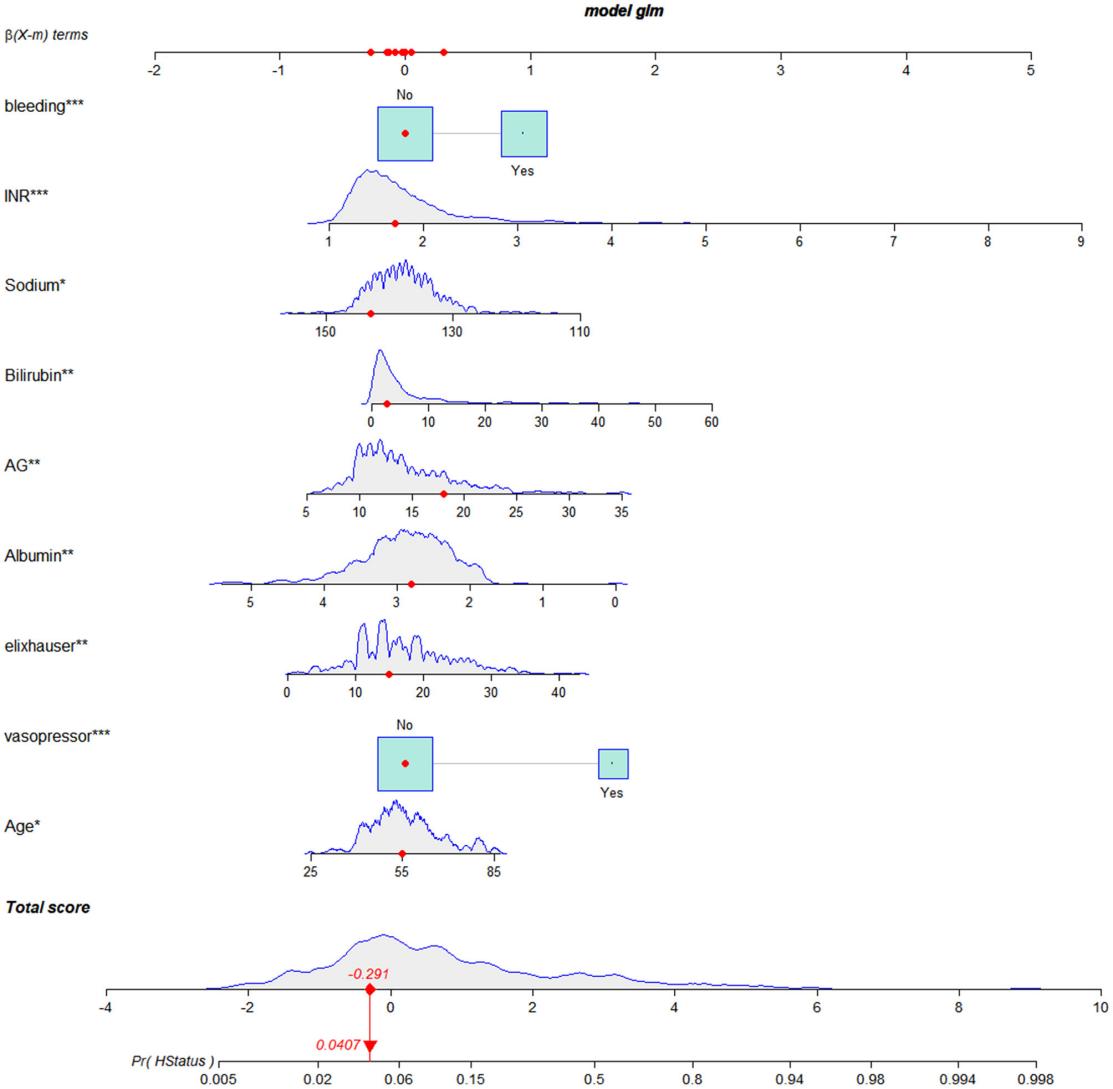
Nomogram for predicting in-hospital death in LCEV patients. LCEV, liver cirrhosis with esophageal; GCS, Glasgow Coma Scale; BUN, blood urea nitrogen; AP, alkaline phosphtaase; INR, international normalized ratio; SpO_2_, percutaneous oxygen saturation; HCC, hepatocellular carcinoma. ^*^P < 0.05, ^**^P < 0.01, ^***^P < 0.001.

We established a nomogram based on the above results that included all of the identified independent prognostic factors to predict in-hospital death in LCEV patients (Figure 2). The nomogram indicates that INR has the greatest influence on the prognosis of LCEV, followed by albumin, bilirubin, AG, sodium, Elixhauser score, vasopressor use, age, and bleeding. We also established a dynamic nomogram (https://xufengshuo.shinyapps.io/LCEV/) to facilitate the application of the model.

### Nomogram Validation

**Figure 3:**
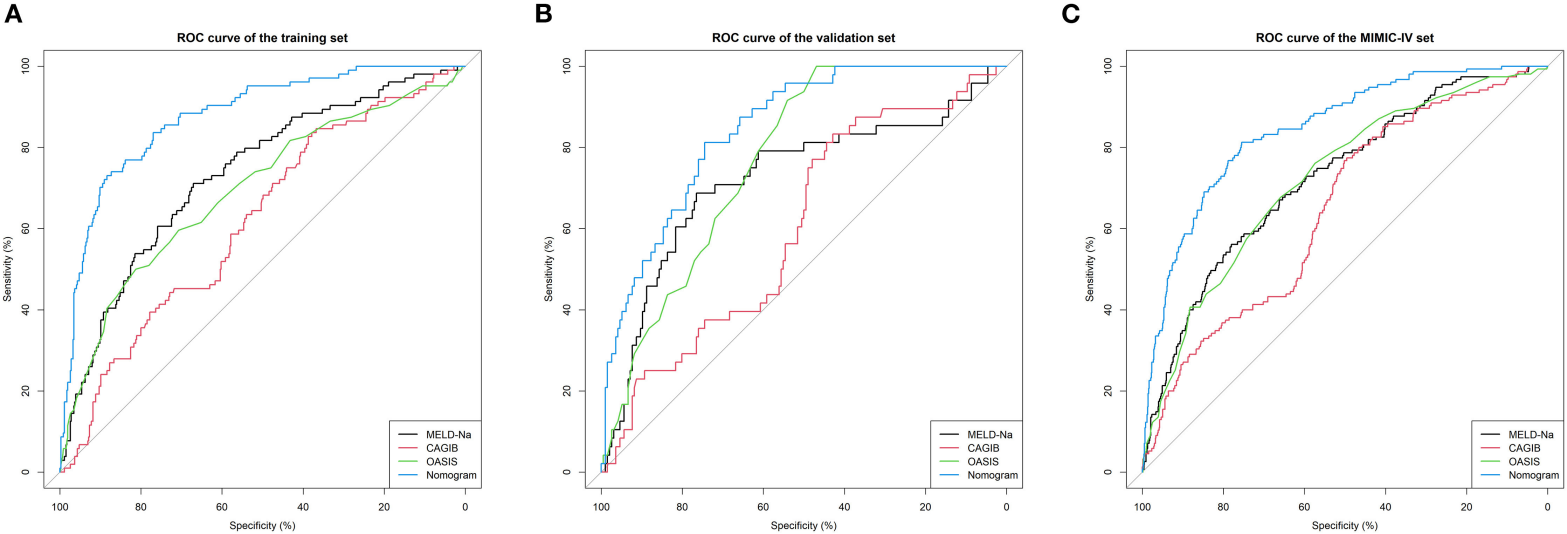
ROC curves. ROC curves were generated to validate the discrimination of the models, by the areas under the ROC curves. **(A–C)** came from the training, validation, and MIMIC-IV cohorts, respectively. LCEV, liver cirrhosis with esophageal; MELD-Na, Model for End-Stage Liver Disease-Na; CAGIB, cirrhosis acute gastrointestinal bleeding; OASIS, Oxford Acute Severity of Illness Score.

We compared the predictive performances for in-hospital death from LCEV of our nomogram and the MELD-Na, CAGIB, and OASIS systems; the results are listed in Table 3. The AUC value of the nomogram was 0.867 (95% CI = 0.832–0.904) in the training cohort, 0.846 (95% CI = 0.790–0.896) in the validation cohort and 0.840 (95% CI = 0.807–0.872) in the MIMIC-IV cohort, which were significantly higher than those for the other scoring systems. The ROC curves are shown in Figure 3. In the training cohort, the optimal cutoff point was 0.250, for which the sensitivity and specificity were 0.884 and 0.731, respectively. In the validation cohort, the optimal cutoff point was 0.145, for which the sensitivity and specificity were 0.745 and 0.813, respectively. And in MIMIC-IV cohort, the optimal cutoff point was 0.139, for which the sensitivity and specificity were 0.755 and 0.813, respectively. Compared with the MELD-Na, CAGIB and OASIS systems, the NRI values were 0.930 (95% CI = 0.666–1.154), 1.192 (95% CI = 1.033–1.383), and 0.857 (95% CI = 0.623–1.154), respectively, in the training cohort, and 1.000 (95% CI = 0.650–1.355), 1.190 (95% CI = 0.920–1.465), and 0.630 (95% CI = 0.288–1.195) in the validation cohort, and 0.689 (95% CI = 0.501–0.915), 0.986 (95% CI = 0.816–1.164), and 0.650 (95% CI = 0.429–0.914) in the MIMIC-IV cohort. The corresponding IDI values were 0.234 (95% CI = 0.184–0.284), 0.335 (95% CI = 0.281–0.389), 0.258 (95% CI = 0.206–0.310), 0.248 (95% CI = 0.173–0.323), 0.332 (95% CI = 0.253–0.411), 0.207 (95% CI = 0.126–0.288), 0.171 (95% CI = 0.134–0.207), 0.250 (95% CI = 0.210–0.289), and 0.170 (95% CI = 0.130–0.211). These values suggest that our nomogram has better discrimination ability and is superior to these commonly used scoring systems.

**Table 3:** Comparison of models in predicting the in-hospital death of LCEV patients.

**Predictive model**		**AUC**	***P*** **-value**	**NRI**	***P*** **-Value**	**IDI**	***P*** **-value**
Training cohort	MELD-Na	0.724 (0.669–0.774)	<0.001	0.930 (0.666–1.154)	<0.001	0.234 (0.184–0.284)	<0.001
	CAGIB	0.611 (0.557–0.667)	<0.001	1.192 (1.033–1.383)	<0.001	0.335 (0.281–0.389)	<0.001
	OASIS	0.662 (0.604–0.731)	<0.001	0.857 (0.623–1.154)	<0.001	0.258 (0.206–0.310)	<0.001
	Nomogram	0.867 (0.832–0.904)					
Validation cohort	MELD-Na	0.699 (0.611–0.787)	0.009	1.000 (0.650–1.355)	<0.001	0.248 (0.173–0.323)	<0.001
	CAGIB	0.601 (0.515–0.692)	<0.001	1.190 (0.920–1.465)	<0.001	0.332 (0.253–0.411)	<0.001
	OASIS	0.766 (0.703–0.831)	0.026	0.630 (0.288–1.195)	<0.001	0.207 (0.126–0.288)	<0.001
	Nomogram	0.846 (0.790–0.896)					
MIMIC-IV dataset	MELD-Na	0.721 (0.679–0.763)	<0.001	0.689 (0.501–0.915)	<0.001	0.171 (0.134–0.207)	<0.001
	CAGIB	0.653 (0.608–0.698)	<0.001	0.986 (0.816–1.164)	<0.001	0.250 (0.210–0.289)	<0.001
	OASIS	0.698 (0.653–0.758)	<0.001	0.650 (0.429–0.914)	<0.001	0.170 (0.130–0.211)	<0.001
	Nomogram	0.840 (0.807–0.872)					

*LCEV, liver cirrhosis with esophageal; AUC, the area under the receiver operating characteristic curve; NRI, net reclassification index; IDI, integrated discrimination improvement; MELD-Na, Model for End-Stage Liver Disease-Na; CAGIB, cirrhosis acute gastrointestinal bleeding; OASIS, Oxford Acute Severity of Illness Score*.

**Figure 4:**
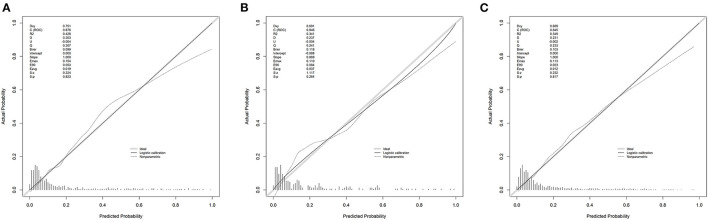
Calibration curves. Calibration curves depict the calibration of the newly established nomogram in terms of the agreement between the predicted probabilities and observed frequencies of the training cohort **(A)**, validation cohort **(B)**, and MIMIC-IV cohort **(C)**.

**Figure 5:**
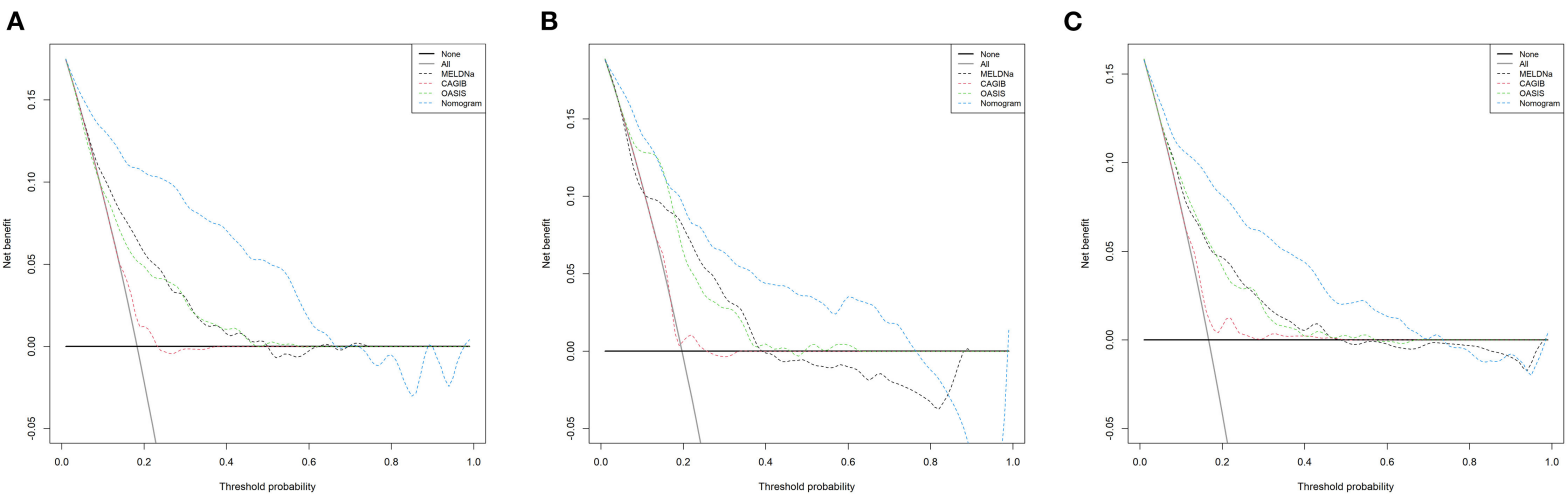
DCA curves of the training cohort **(A)**, validation cohort **(B)**, and MIMIC-IV cohort **(C)**. In the figure, the abscissa is the threshold probability, the ordinate is the net benefit rate. The horizontal one indicates that all samples are negative and all are not treated, with a net benefit of zero. The oblique one indicates that all samples are positive. The net benefit is a backslash with a negative slope. LCEV, liver cirrhosis with esophageal; MELD-Na, Model for End-Stage Liver Disease-Na; CAGIB, cirrhosis acute gastrointestinal bleeding; OASIS, Oxford Acute Severity of Illness Score.

Figure 4 shows the calibration curves for the nomogram. The calibration curves of the training and validation cohorts were close to the leading diagonal. And the results of Hosmer-Lemeshow test were not statistically significant (chi-square = 7.403 and *P* = 0.595 for the training cohort, chi-square = 7.630 and *P* = 0.572 for the validation cohort, chi-square = 6.497 and *P* = 0.689 for the MIMIC-IV cohort). All of these indicated that our nomogram provided a good fit to the available data. Finally, we plotted DCA curves to illustrate the clinical value of the nomogram and compared it with those of OASIS, MELD-Na, and CAGIB systems (Figure 5). When the threshold probability was between 0.1 and 0.7 (in either cohort), clinical interventions guided by the nomogram had greater net benefits than the other scoring systems.

Further, we generated ROC curves for each continuous variable among independent prognostic factors, as shown in Supplementary Figure 2. The AUC of all variables were higher than 0.5, indicating that their inclusion in the predictive model was reliable. In addition, we also performed subgroup analysis, as shown in Supplementary Figure 3. The AUC of the nomogram for the non-bleeding cohort and the bleeding cohort were 0.866 (95% CI = 0.835–0.894) and 0.847 (95% CI = 0.818–0.873), both higher than the other scoring systems. The results showed that in different subgroups, the nomogram has good predictive performance.

## Discussion

Liver cirrhosis results from the development of various acute and chronic liver diseases. Liver cirrhosis from any cause can lead to either obstruction of or increased blood flow in the portal vein, leading to portal hypertension, or lateral circulation open. The main cause of esophageal varices is portal hypertension. LCEV is a common critical complication of decompensated cirrhosis. The prognosis of LCEV prognosis remains poor despite the development of various treatment methods. It is therefore very important to develop a convenient and effective prognostic model that stratifies the risk of LCEV patients in order to guide treatments (9).

The MIMIC-III and MIMIC-IV databases contain a large number of clinical diagnoses and treatment data for critically ill patients, thereby providing effective samples for clinicians to conduct scientific research. This study used the MIMIC-III and MIMIC-IV databases to extensively explore independent predictors of in-hospital death in LCEV patients, which include age, vasopressor use, Elixhauser score, albumin, AG, bilirubin, sodium, INR, and bleeding. We applied these factors to a logistic regression model and generated a nomogram to display it. In addition, we created a Web-based dynamic nomogram to facilitate its clinical application. To the best of our knowledge, this is the first nomogram to be applied to LCEV patients. Notably, the vital signs used in this study were the mean values from the first 24 h of ICU admission, and laboratory test results used were the first obtained after an ICU admission. The nomogram was therefore not applicable to patients who died or were discharged within 24 h of ICU admission.

This study found that age, vasopressor use, Elixhauser score, albumin, AG, bilirubin, sodium, INR, and bleeding were important prognostic factors for LCEV, which is consistent with the findings of other studies. These factors are also commonly used indicators in many severity scoring systems for cirrhosis, such as MELD-Na and CAGIB.

Age has been proven to be the main factor for the poor prognosis of various diseases (24). The reason is that with age, the body's immunity will inevitably decrease (25, 26). Moreover, the function of the organs will decline. For example, elderly patients have reduced gastrointestinal digestive function, limited ability to absorb nutrients, and are extremely prone to malnutrition, which will adversely affect the prognosis of patients. In addition, elderly patients have more comorbidities than younger patients, so the situation will be more serious. The impact of Elixhauser score on the prognosis also illustrates this point. It is a comorbidity scoring system based on the number and severity of the disease that a patient suffers from and quantifies their comorbidities. As the number of comorbidities increases, the patient's prognosis becomes worse (27).

It can be seen from the nomogram that the INR occupies a greater weight, and as the INR increases, the patient's prognosis becomes worse. INR is an indicator of blood coagulation function. The reason for its prolongation is that the patient enters the decompensated phase of liver cirrhosis, liver function continues to deteriorate, prothrombin synthesis is impaired, which leads to prolonged PT. At the same time, the activation of mononuclear phagocytes caused by spleen enlargement increases platelet destruction, which will reduce blood coagulation function (1, 28). Therefore, patients with liver cirrhosis often have nasal cavity, gum bleeding, skin and mucous membrane petechiae and gastrointestinal bleeding, etc., which are also related to the above-mentioned mechanisms such as reduction of hepatic coagulation factors and hypersplenism, reflecting that the patient is in decompensation, leading to poor prognosis (29).In addition, the use of vasopressor is also one of the factors of poor prognosis for patients, which means that the patient has already experienced a drop-in blood pressure, and drugs are needed to improve vascular function and microcirculation blood perfusion. The reason may be that the blood volume is decreased due to heavy bleeding in the digestive system, or the patient has spontaneous peritonitis (30), or portal hypertension reduces the intestinal mucosal barrier function, and bacteremia caused by bacteria in the intestinal cavity entering the blood circulation. Under the action of inflammatory mediators, blood volume decreases, and blood vessel elasticity decreases (31).

Albumin and bilirubin are also important indicators that reflect liver function. Studies have shown that low serum albumin is common in liver cirrhosis and is related to reduced survival rates (32). Changes in bilirubin levels often indicate liver dysfunction in patients with liver cirrhosis, which is closely related to a poor prognosis (33).

In recent years, many studies have found the clinical value of the anion gap (AG) in assessing the prognosis of the disease (34). For instance, in patients with acute myocardial infarction, compared with patients with normal AG, the hospitalization rate of patients with high AG increased, and the mortality rate within 1 week of admission increased (35). The most common disease with elevated AG is metabolic acidosis, which means the overproduction of organic acids, such as the accumulation of lactic acid, the production of toxins from keto acids, and metabolic acidosis caused by uremia. Patients with elevated AG are accompanied by severe electrolyte abnormalities, and this is related to the severity of the disease (36).Serum sodium is an indicator in the MELD-Na system. Most scholars believe that hyponatremia is associated with portal hypertension, and that integrating this indicator in the MELD system improves its prediction accuracy. Our study also similarly concluded that serum sodium is a protective factor in the prognosis of LCEV patients. On the one hand, there are sodium in the calculation formula of AG, and the increase in serum sodium also indirectly reflects the increase in AG. Another aspect, sodium can also be an indicator of cirrhosis progression. The causes of hyponatremia in cirrhosis include obvious liver damage, Na+-K+-ATP dysfunction, and reduced cellular release of Na+; aldosterone, antidiuretic hormone, atrial natriuretic peptide, and other hormones not being metabolized by the liver, resulting in water retention and dilution causing low sodium levels; and the rapid release of large amounts of AC, excessive diuresis, vomiting, diarrhea, and long-term low-salt diets, causing sodium loss (37).

A nomogram is commonly used method for presenting a model that combines important prognostic factors and specific endpoints to quantitatively assess the prognostic risk of individual patients. Our nomogram contains a small number of effective and readily available prognostic factors for LCEV patients, making it easy to use. As shown in Figure 2, a score was assigned to each characteristic of a patient, and the scores are then summed to obtain an overall score, which corresponds to the in-hospital death risk. We also generated a more user-friendly dynamic nomogram. In order to confirm the validity of our nomogram, we used multiple indicators in the training, validation and MIMIC-IV cohorts to compare its performance with MELD-Na, CAGIB, and OASIS systems in predicting the prognosis of LCEV patients. As is evident from the section Results, our nomogram is superior to these other scoring systems in terms of differentiation, calibration, and clinical application.

There are inevitable limitations to our study. First, because the MIMIC is a single-center database, our study had selection bias and restricted generalizability. Although the new model based on MIMIC-III has achieved good validation results in MIMIC-IV, it still needs to be validated in datasets other than MIMIC. Second, many potential prognostic factors were not included in our model, which reduced the accuracy of the nomogram predictions. A nomogram obviously does not provide completely accurate prognosis predictions, and so should only be used as a reference by clinicians. Third, our study was based on a retrospective cohort, and so the nomogram needs further prospective validation before being considered for clinical application.

## Conclusion

We established the first prognostic nomogram for predicting the in-hospital death of LCEV patients based on the MIMIC database. The nomogram is easy to use, performs well, and can be used to guide clinical practice; however, further external prospective validation is needed.

## Data Availability Statement

The datasets presented in this study can be found in online repositories. The names of the repository/repositories and accession number(s) can be found below: https://physionet.org/content/mimiciii-demo/1.4/ and https://physionet.org/content/mimiciv/1.0/.

## Ethics Statement

The use of the MIMIC database was approved by the Institutional Review Board of the Beth Israel Deaconess Medical Center and Massachusetts Institute of Technology, and all patient information in the database is anonymous, so informed consent was not required (29, 30). We completed the online course and examination to gain access to the database (Record ID: 38455175).

## Author Contributions

FX and LZ analyzed the data and wrote the paper. ZW and DH collected the data. CL and SZ checked the integrity of the data and the accuracy of the data analysis. FX, HY, and JL designed the study and revised the paper. All authors read and approved the final manuscript.

## Funding

This study was supported by the National Social Science Foundation of China (grant/award no. 16BGL183).

## Conflict of Interest

The authors declare that the research was conducted in the absence of any commercial or financial relationships that could be construed as a potential conflict of interest.

## Publisher's Note

All claims expressed in this article are solely those of the authors and do not necessarily represent those of their affiliated organizations, or those of the publisher, the editors and the reviewers. Any product that may be evaluated in this article, or claim that may be made by its manufacturer, is not guaranteed or endorsed by the publisher.

## Supplementary Material

Supplementary Figure 1 Data missing before multiple imputation. **(A)** MIMIC-III database; **(B)** MIMIC-IV database. DB, diabetes; HCC, hepatocellular carcinoma; AC, ascites; HE, hepatic encephalopathy; OASIS, Oxford Acute Severity of Illness Score; Mechvent, mechanical ventilation; RRT, renal replacement treatment; WBC, white blood cells; RBC, red blood cells; RDW, RBC distribution width; MCV, mean corpuscular volume; MCH, mean corpuscular hemoglobin; BUN, blood urea nitrogen; AG, anion gap; PTT, partial prothrombin time; PT, prothrombin time; INR, international normalized ratio; GCS, Glasgow Coma Scale; SpO_2_, percutaneous oxygen saturation; MBP, mean blood pressure; AST, aspartate aminotransferase; ALT, alanine aminotransferase; AP, alkaline phosphtaase; LD, lactate dehydrogenase.

Supplementary Figure 2 ROC curves for all independent predictors. AG, anion gap; INR, international normalized ratio.

Supplementary Figure 3 ROC curves for **(A)** non-bleeding cohort and **(B)** bleeding cohort. MELD-Na, Model for End-Stage Liver Disease-Na; CAGIB, cirrhosis acute gastrointestinal bleeding; OASIS, Oxford Acute Severity of Illness Score.

Supplementary Table 1 Baseline demographic and laboratory characteristics of LCEV patients in MIMIC-IV database.
