# Supplementary figures and images for: A New Scoring System for Predicting In-hospital Death in Patients Having Liver Cirrhosis With Esophageal Varices
Source: Front Med (Lausanne). 2021 Oct 11;8:678646. doi: 10.3389/fmed.2021.678646 (PMC8542681; doi:10.3389/fmed.2021.678646)

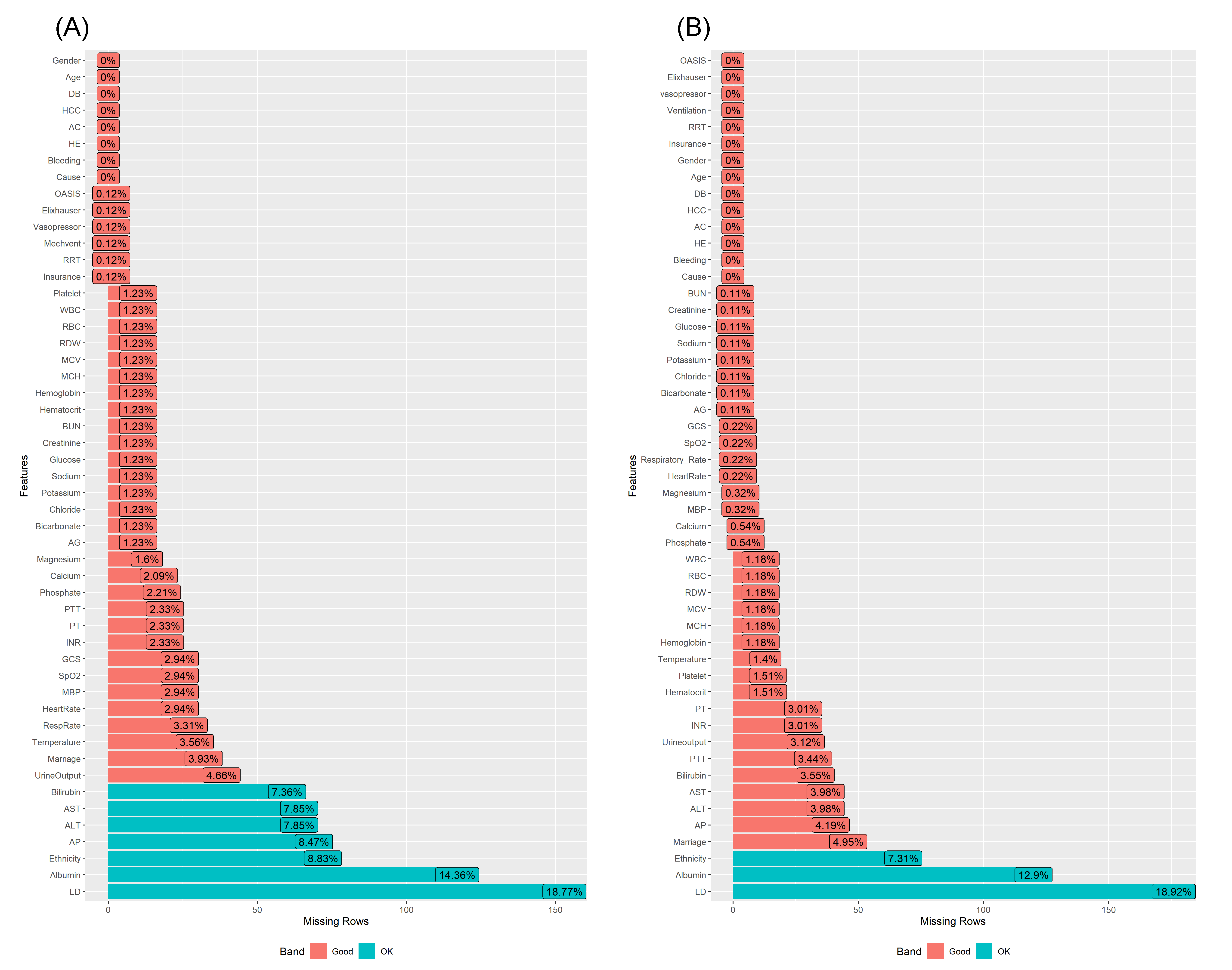

Supplement: Supplementary Figure 1 — Data missing before multiple imputation. (A) MIMIC-III database; (B) MIMIC-IV database. DB, diabetes; HCC, hepatocellular carcinoma; AC, ascites; HE, hepatic encephalopathy; OASIS, Oxford Acute Severity of Illness Score; Mechvent, mechanical ventilation; RRT, renal replacement treatment; WBC, white blood cells; RBC, red blood cells; RDW, RBC distribution width; MCV, mean corpuscular volume; MCH, mean corpuscular hemoglobin; BUN, blood urea nitrogen; AG, anion gap; PTT, partial prothrombin time; PT, prothrombin time; INR, international normalized ratio; GCS, Glasgow Coma Scale; SpO2, percutaneous oxygen saturation; MBP, mean blood pressure; AST, aspartate aminotransferase; ALT, alanine aminotransferase; AP, alkaline phosphtaase; LD, lactate dehydrogenase. [file Image_1.TIF]

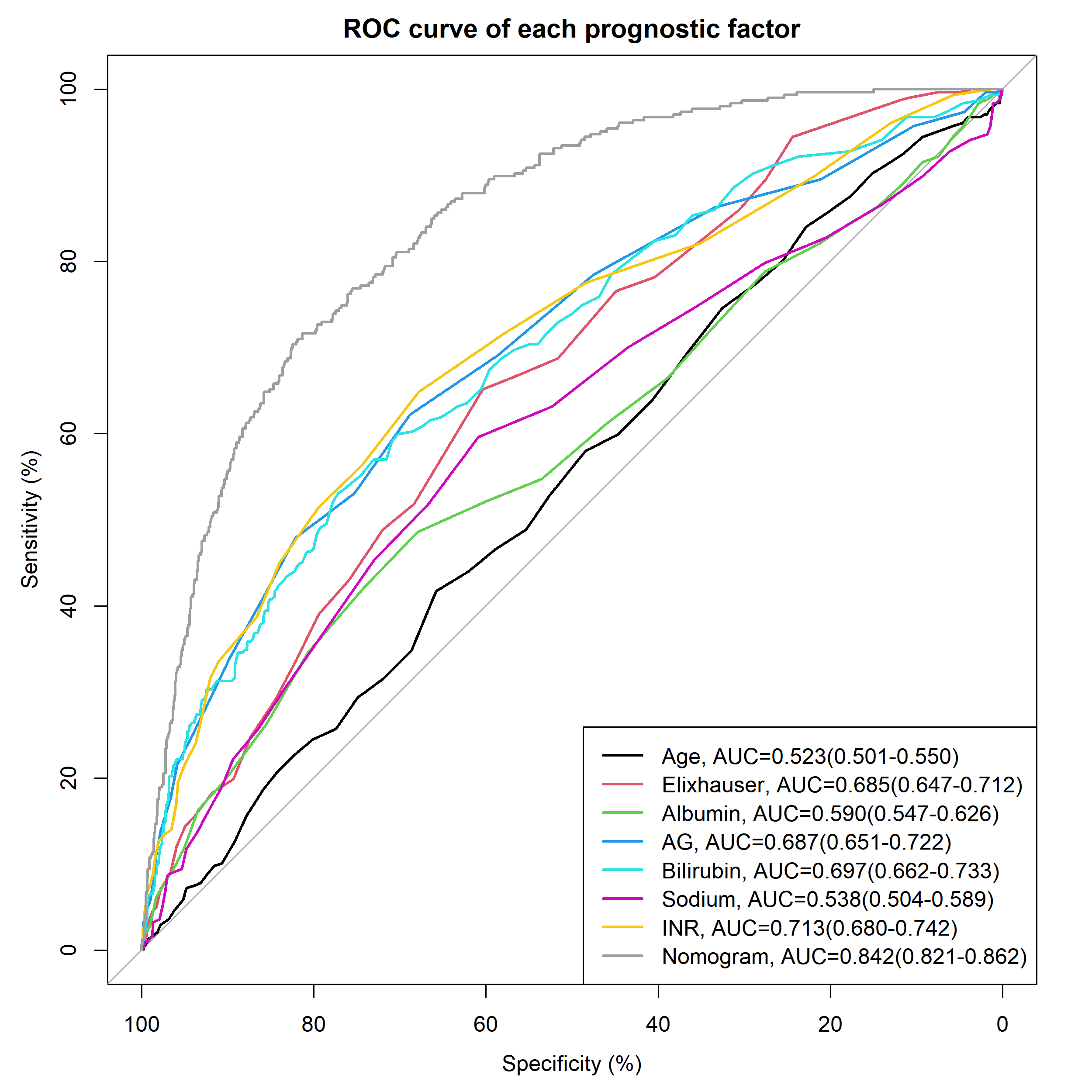

Supplement: Supplementary Figure 2 — ROC curves for all independent predictors. AG, anion gap; INR, international normalized ratio. [file Image_2.TIFF]

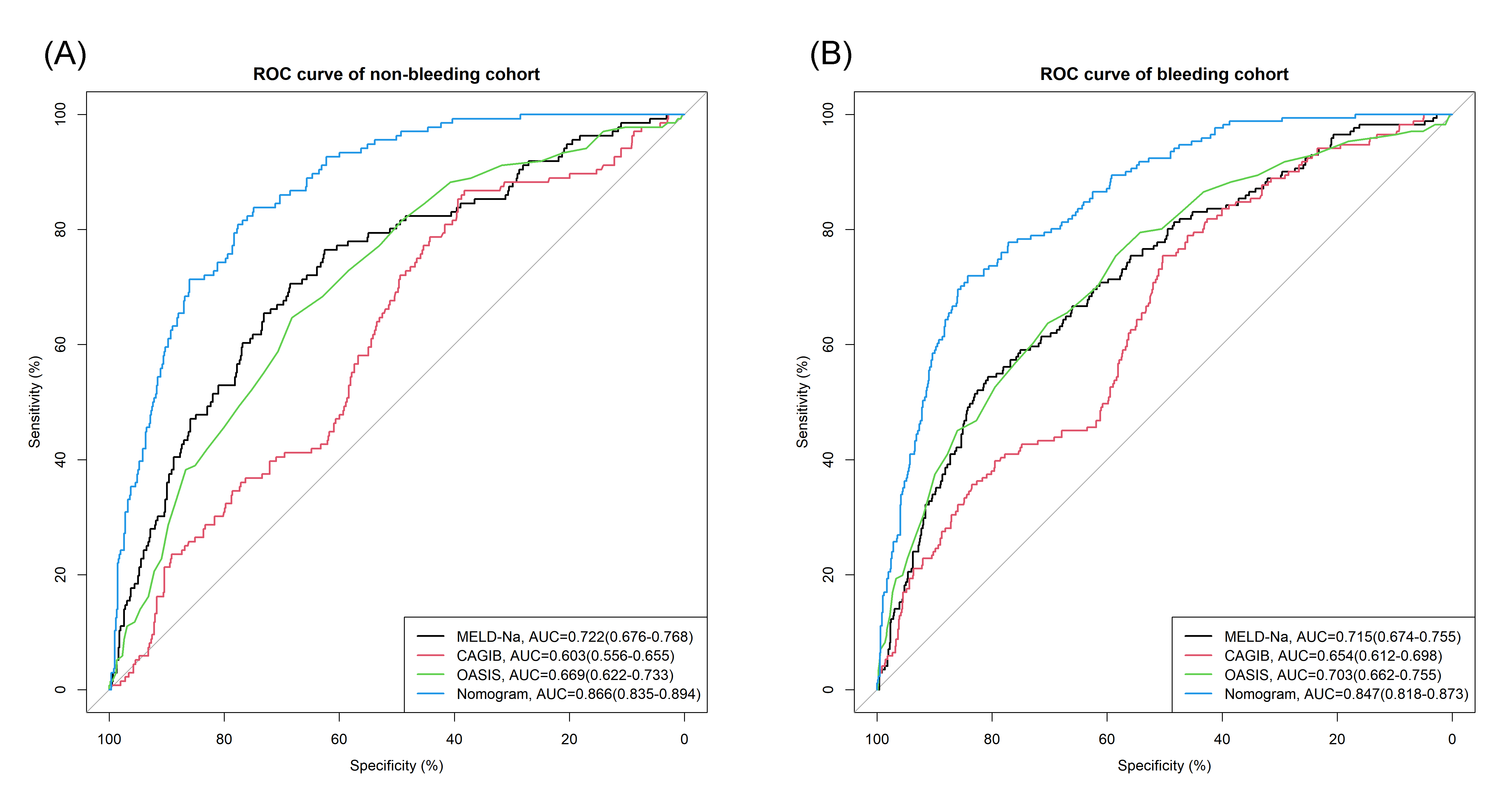

Supplement: Supplementary Figure 3 — ROC curves for (A) non-bleeding cohort and (B) bleeding cohort. MELD-Na, Model for End-Stage Liver Disease-Na; CAGIB, cirrhosis acute gastrointestinal bleeding; OASIS, Oxford Acute Severity of Illness Score. [file Image_3.TIF]
