# Supplementary material for: A New Scoring System for Predicting In-hospital Death in Patients Having Liver Cirrhosis With Esophageal Varices
Source: Front Med (Lausanne). 2021 Oct 11;8:678646. doi: 10.3389/fmed.2021.678646 (PMC8542681; doi:10.3389/fmed.2021.678646)
Supplement: Supplementary Table 1 — Baseline demographic and laboratory characteristics of LCEV patients in MIMIC-IV database. [file Table_1.DOCX]

Table S1. Baseline demographic and laboratory characteristics of LCEV patients in MIMIC-IV database.

| **Variables** | **MIMIC-IV dataset** |
| --- | --- |
| **N** | 930 |
| **Cause, n(%)** |  |
| Cholestasis or alcoholic | 509 (54.7) |
| Other | 421 (45.3) |
| **Bleeding, n(%)** |  |
| No | 285 (30.6) |
| Yes | 645 (69.4) |
| **HE, n(%)** |  |
| No | 712 (76.6) |
| Yes | 218 (23.4) |
| **AC, n(%)** |  |
| No | 456 (49.0) |
| Yes | 474 (51.0) |
| **HCC, n(%)** |  |
| No | 833 (89.6) |
| Yes | 97 (10.4) |
| **DB, n(%)** |  |
| No | 647 (69.6) |
| Yes | 283 (30.4) |
| **Age(Year) (median [IQR])** | 57.0 [51.0,64.0] |
| **Gender, n(%)** |  |
| Male | 614 (66.0) |
| Female | 316 (34.0) |
| **Marrital Status, n(%)** |  |
| Married | 386 (41.5) |
| Unmarried | 377 (40.5) |
| Other | 167 (18.0) |
| **Ethnicity, n(%)** |  |
| White | 669 (71.9) |
| Black | 94 (10.1) |
| Other | 167 (18.0) |
| **Insurance, n(%)** |  |
| Medicaid | 178 (19.1) |
| Medicare | 305 (32.8) |
| Other | 447 (48.1) |
| **Heart Rate(min-1) (median [IQR])** | 83.3 [71.4,95.8] |
| **MBP(mmHg) (median [IQR])** | 73.3 [67.3,81.9] |
| **Respiratory Rate(min-1) (median [IQR])** | 17.4 [15.1,20.2] |
| **Temperature(℃) (median [IQR])** | 36.8 [36.5,37.0] |
| **SpO2(%) (median [IQR])** | 97.4 [96.0,98.7] |
| **24-hour Urine Output(mL) (median [IQR])** | 775.0 [125.0,1261.3] |
| **ALT(IU/L) (median [IQR])** | 33.0 [22.0,55.0] |
| **AST(IU/L) (median [IQR])** | 63.0 [41.0,109.0] |
| **Albumin(g/dL) (median [IQR])** | 2.9 [2.5,3.3] |
| **Bilirubin(mg/dL) (median [IQR])** | 2.6 [1.3,5.7] |
| **AP(IU/L) (median [IQR])** | 100.5 [72.0,147.0] |
| **AG(mEq/L) (median [IQR])** | 13.0 [11.0,16.0] |
| **Bicarbonate(mEq/L) (median [IQR])** | 23.0 [19.0,25.0] |
| **Phosphate(mg/dL) (median [IQR])** | 3.4 [2.9,4.2] |
| **Chloride(mEq/L) (median [IQR])** | 105.0 [99.2,109.0] |
| **Calcium(mg/dL) (median [IQR])** | 8.2 [7.7,8.7] |
| **Magnesium(mg/dL) (median [IQR])** | 1.9 [1.7,2.1] |
| **Potassium(mEq/L) (median [IQR])** | 4.2 [3.8,4.7] |
| **Sodium(mEq/L) (median [IQR])** | 137.0 [133.0,140.0] |
| **Glucose(mg/dL) (median [IQR])** | 242.0 [195.0,318.8] |
| **LD(IU/L) (median [IQR])** | 117.0 [97.0,150.0] |
| **Creatinine(mg/dL) (median [IQR])** | 1.1 [0.7,1.7] |
| **BUN(mg/dL) (median [IQR])** | 24.5 [15.0,41.0] |
| **Hematocrit(%) (median [IQR])** | 28.9 [25.3,32.9] |
| **Hemoglobin(g/dL) (median [IQR])** | 9.6 [8.4,11.1] |
| **MCH(pg) (median [IQR])** | 31.8 [29.7,34.0] |
| **MCV(fL) (median [IQR])** | 94.0 [90.0,101.0] |
| **RDW(%) (median [IQR])** | 16.9 [15.4,18.7] |
| **RBC(m/uL) (median [IQR])** | 3.0 [2.6,3.5] |
| **WBC(k/uL) (median [IQR])** | 6.9 [4.4,10.4] |
| **Platelet(k/uL) (median [IQR])** | 88.0 [60.0,133.0] |
| **INR (median [IQR])** | 1.6 [1.4,2.0] |
| **PT(s) (median [IQR])** | 17.7 [15.2,21.3] |
| **PTT(s) (median [IQR])** | 36.8 [32.0,44.8] |
| **RRT, n(%)** |  |
| No | 827 (88.9) |
| Yes | 103 (11.1) |
| **Mechvent, n(%)** |  |
| No | 449 (48.3) |
| Yes | 481 (51.7) |
| **Vasopressor, n(%)** |  |
| No | 724 (77.8) |
| Yes | 206 (22.2) |
| **Elixhauser (median [IQR])** | 18.0 [13.0,23.0] |
| **GCS (median [IQR])** | 15.0 [15.0,15.0] |
| **OASIS (median [IQR])** | 31.0 [25.0,38.0] |
| **MELD-Na (median [IQR])** | 10.3 [0.3,23.8] |
| **CAGIB (median [IQR])** | -4.1 [-4.2,-3.2] |
| **In-hospital death, n(%)** |  |
| Alive | 775 (83.3) |
| Dead | 155 (16.7) |

Abbreviations: LCEV, liver cirrhosis with esophageal; HE, hepatic encephalopathy; AC, ascites; HCC, hepatocellular carcinoma; DB, diabetes; IQR, interquartile-range; MBP, mean blood pressure; SpO2, percutaneous oxygen saturation; ALT, alanine aminotransferase; AP, alkaline phosphtaase; AG, anion gap; AST, aspartate aminotransferase; LD, lactate dehydrogenase; BUN, blood urea nitrogen; MCH, mean corpuscular hemoglobin; MCV, mean corpuscular volume; RBC, red blood cells; WBC, white blood cells; RDW, RBC distribution width; INR, international normalized ratio; PT, prothrombin time; PTT, partial prothrombin time; RRT, renal replacement treatment; Mechvent, mechanical ventilation; GCS, Glasgow Coma Scale; OASIS, Oxford Acute Severity of Illness Score; MELD-Na, Model for End-Stage Liver Disease-Na; CAGIB, cirrhosis acute gastrointestinal bleeding.
